# Langerhans Cells—Revising Their Role in Skin Pathologies

**DOI:** 10.3390/jpm12122072

**Published:** 2022-12-15

**Authors:** Monica Neagu, Carolina Constantin, Gheorghita Jugulete, Victor Cauni, Sandrine Dubrac, Attila Gábor Szöllősi, Sabina Zurac

**Affiliations:** 1 Immunology Department, “Victor Babes” National Institute of Pathology, 050096 Bucharest, Romania; 2 Department of Pathology, Colentina Clinical Hospital, 020125 Bucharest, Romania; 3 Faculty of Biology, University of Bucharest, 76201 Bucharest, Romania; 4 Department of Infectious Diseases, “Carol Davila” University of Medicine and Pharmacy, 050474 Bucharest, Romania; 5 Clinical Section IX—Pediatrics, “Prof. Dr. Matei Balş” National Institute for Infectious Diseases, 050474 Bucharest, Romania; 6 Department of Urology, Colentina University Hospital, 050474 Bucharest, Romania; 7 Department of Dermatology, Venereology and Allergology, Medical University of Innsbruck, 6020 Innsbruck, Austria; 8 Department of Immunology, Faculty of Medicine, University of Debrecen, 4032 Debrecen, Hungary; 9 Department of Pathology, “Carol Davila” University of Medicine and Pharmacy, 050474 Bucharest, Romania

**Keywords:** Langerhans cell, non-melanoma skin cancers, cutaneous melanoma, immune cells, inflammation, therapy

## Abstract

Langerhans cells (LCs) constitute a cellular immune network across the epidermis. Because they are located at the skin barrier, they are considered immune sentinels of the skin. These antigen-presenting cells are capable of migrating to skin draining lymph nodes to prime adaptive immune cells, namely T- and B-lymphocytes, which will ultimately lead to a broad range of immune responses. Moreover, LCs have been shown to possess important roles in the anti-cancer immune responses. Indeed, the literature nicely highlights the role of LCs in melanoma. In line with this, LCs have been found in melanoma tissues where they contribute to the local immune response. Moreover, the immunogenic properties of LCs render them attractive targets for designing vaccines to treat melanoma and autoimmune diseases. Overall, future studies will help to enlarge the portfolio of immune properties of LCs, and aid the prognosis and development of novel therapeutic approaches to treating skin pathologies, including cancers.

## 1. Introduction

**Figure 1:**
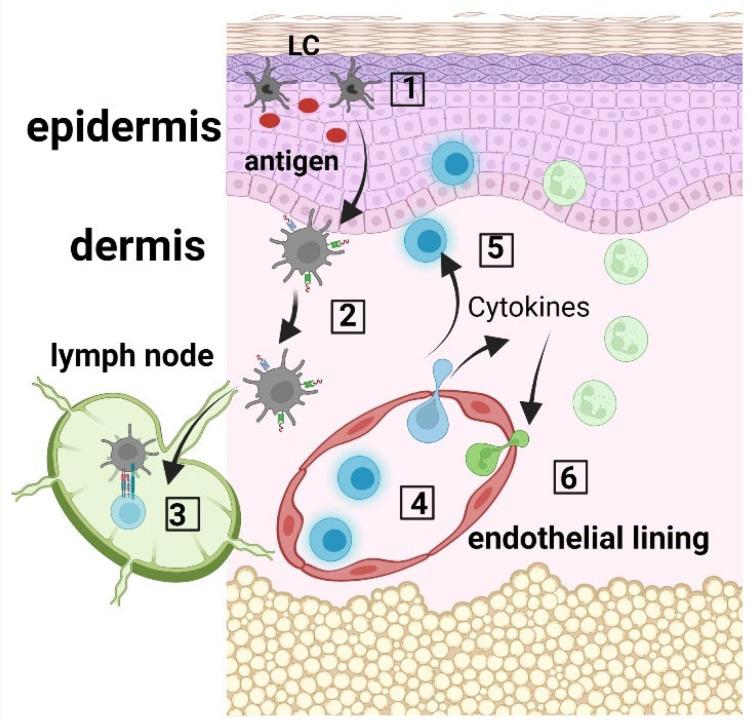
LC immune network. (**1**) Langerhans cells (LCs) within the epidermis take up antigen; (**2**) LCs internalize and process the antigen, and present the produced peptides on their surface via MHC molecules; (**3**) LCs migrate to the skin draining lymph nodes where they prime CD45RA^+^ naïve T cells; (**4**) Activated T cells migrate to the skin, where they produce cytokines and chemokines (**5**), which recruit innate immune cells, including neutrophils from the blood circulation (**6**).

Langerhans cells (LCs) are ontogenetically tissue resident macrophages that are functionally more similar to dendritic cells (DCs). In their steady state, they are found mainly in the epidermis, and after activation, they can migrate to skin draining lymph nodes where they play the role of antigen presenting cells (APCs). LCs display important roles in both the stimulation and suppression of adaptive immune responses. After uptake, LCs process antigens into peptides that they then present via MHC molecules to either naïve T cells in the skin draining lymph nodes or to memory T cells in the skin. LCs can activate T cells to produce various adaptive immune responses, including Th1, Th2, Th17 immune responses, as well as immunosuppression via the expansion of regulatory T cells (Tregs). Figure 1 depicts the functions of LCs in the skin.

In the steady state, the renewal of LCs in the epidermis is mediated via the local proliferation of skin resident LCs. Indeed, mature LCs residing in the skin possess an intrinsic ability to enter a cell division cycle, when required, hence allowing the replenishment of the LC population in the epidermis [1]. LCs constitute an immune network across the epidermis and, in turn, serve as skin immune sentinels [2]. Thus, in inflammatory conditions, LCs continuously migrate to the skin draining lymph nodes [3]. When a mass exodus of LCs leads to their numbers being depleted, LC replenishment is ensured by two successive waves of blood-derived cells, i.e., monocytes [4], followed by a myeloid precursor [5,6].

LCs are characterized by the expression of langerin and the presence of Birbeck granules in both mice and humans. However, in vivo mouse experiments have shown that langerin is not a specific marker of LCs because other subsets of DCs, which are located in extra-cutaneous and non-lymphoid tissues, also express langerin [7]. Furthermore, subsets of DCs found in most connective tissues, including the dermis, express langerin. Langerin^+^ DCs are also positive for CD103 and are CD11b^low^, in contrast to LCs [8]. LCs express Langerin/CD207, MHC class II, E-Cadherin, EpCAM (Epithelial Cell Adhesion Molecule)/TROP1, Integrin alpha X/CD11c, and human LCs additionally express CD1a, whereas mouse LCs express CD11b^+^, CD205^+^ and F4/80^+^ [6].

Single-cell RNASeq data and mass spectrometry flow cytometry have recently shown that LCs are not a homologous population in the skin [9,10,11]. These results show that LCs can be subcategorized into two major classes (LC1 and LC2); these cell types are either localized to the skin, or are activated and migratory cell types in transit from the epidermis. In general, LC1 are specialized toward antigen uptake and cytokine secretion related to innate immunity, while adaptive and tolerogenic responses are better initiated by LC2 cells. These subsets have only recently been defined; therefore, their exact role in the development of skin pathologies has not been investigated in detail, but it might account for some of the discrepancies encountered when investigating the role of LCs in skin diseases.

In this review, we outline the main role of LCs in skin inflammation, autoimmune diseases and cancers, and emphasize therapeutic approaches targeting LCs.

## 2. Inflammation and Inflammation-Mediated Skin Pathologies

### 2.1. Role of Langerhans Cells in Wound Healing

Many skin injuries, such as wounds, provoke the migration of LCs out of the epidermis. In this context, the repopulation of the tissue is first ensured by circulating monocytes [6], recruited to the epidermis via the expression of chemokine receptors (CCR)-2 and -6. Then, the full replenishment of LCs will progressively be ensured by resident LCs expressing Langerin, CD24, and EpCAM [6]. The full acquisition of proper functionality of these immature LCs requires the keratinocyte-derived interleukin (IL)-34 [12]. In a wound environment, keratinocytes up-regulate MICA B (Major histocompatibility complex class I-related chain B), a NKG2D ligand (lymphocyte activation receptor natural killer group 2D), which results in LC migration, and the recruitment of αβ T cells to the epidermis [13,14]. The continuous cross-talk between keratinocytes and LCs is critically involved in the LC migratory process after skin wounding [15]. This inter-cellular communication is guaranteed by pro-inflammatory cytokines, [e.g., IL-1, granulocyte macrophage colony stimulating factor (GM-CSF), tumor necrosis factor (TNF-α)], which are involved in LC migration [16]. In the inflammatory phase of tissue remodeling, LCs repopulate the epidermis [17]. This is likely facilitated by TNF-α, which up-regulates the expression of surface adhesion molecules [18]. In patients diagnosed with diabetes, cutaneous repair is delayed. When glycaemia is normalized in such patients, the number of LCs increases in the epidermis. In diabetic foot ulcers, therapy can enhance the number of LCs in the epidermis, which is associated with better healing, emphasizing the importance of LCs in the regenerative capacity of the skin after wounding [19].

However, the detailed role of LCs in wound healing is not yet fully deciphered and a better understanding of the importance of LCs in such processes will open new therapeutic avenues in wound-induced acute or chronic inflammation [20].

### 2.2. Role of Langerhans Cells in Psoriasis

There are abundant results reporting on the role of LCs in psoriasis [2,21]. In experimental mouse models [22], it was demonstrated that the IL-23/Th17 axis is involved in the psoriasis-like inflammation [23]. LCs [24] or cDCs [25] have been shown to be, alternatively, the major source of IL-23 in this animal model (i.e., topical application of Imiquimod). The animal model of induced psoriasis using the topical application of Imiquimod is a commonly used psoriasis-mouse model, where, after several applications of the compound on the shaved skin psoriatic, lesions appear. These skin inflammation lesions are severe and animals can be scored as psoriasis patients using individual PASI scores (e.g., erythema, thickening and skin scaling) [22]. The fact that published reports show discrepancies regarding the IL-23 cellular source, is explained by experimental details regarding the model, with psoriasis-like inflammation induced on the ear versus on the back of animals. CD1a is a surface molecule expressed at the cell surface of LCs, which presents lipid antigens. When CD1a is over-expressed, symptoms of psoriasis are aggravated in the skin. In line with this, in psoriatic patients, inflammation and amounts of cytokines in the skin, especially TNF-α, IL-1β [26,27] and IL-17α [28], are lowered by treatment with anti-CD1a antibodies [29].

**Figure 2:**
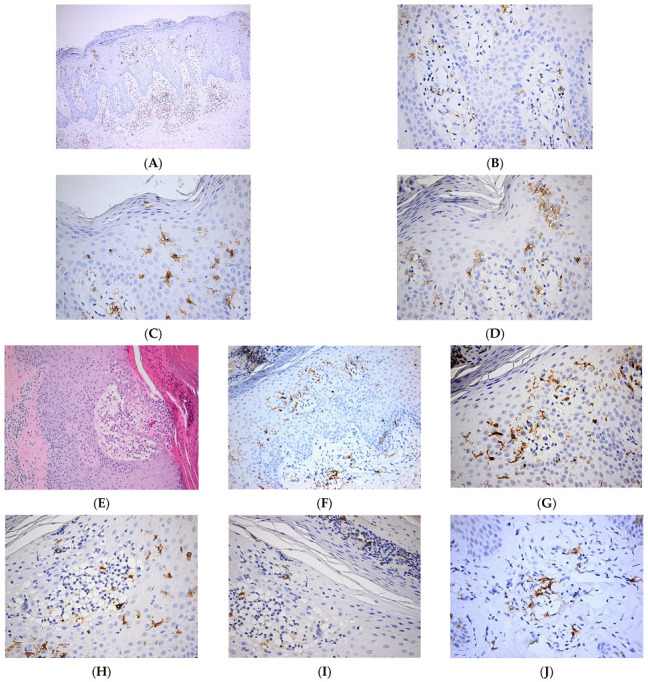
Langerhans cell distribution in psoriatic skin lesions. (**A**). Numerous Langerhans cells are present both within the epidermis and dermis. CD1a × 100. (**B**). Numerous Langerhans cells with perivascular distribution in papillary dermis. CD1a × 400. (**C**). Frequent intraepidermal Langerhans cells with diffuse distribution in the whole epidermal layer. Occasional Langerhans cells within the parakeratotic layer. CD1a × 400. (**D**). Frequent intraepidermal Langerhans cells with a predominantly basal/suprabasal location. Few Langerhans cells present in the corneous layer. CD1a × 400; (**E**–**J**). Pustulous psoriasis. (**E**). Thick epidermis with subcorneous vesicle filled by serum containing neutrophils; suprajacent agranulosis; marked parakeratosis with neutrophilic abscess; keratinocytic proliferation HE × 100. (**F**). Numerous Langerhans cells within the epidermis and papillary dermis. CD1a × 100. (**G**). Numerous Langerhans cells with predominantly basal/suprabasal location. CD1a × 400. (**H**). Numerous intraepidermal Langerhans cells are present both within the subcorneous vesicle and in the adjacent epidermis. CD1a × 400. (**I**). Frequent intraepidermal Langerhans cells present in the parakeratotic corneous layer. CD1a × 400. (**J**). Perivascular Langerhans cells in papillary dermis. CD1a × 400.

In a recent work, it was shown that diminished secretion of IL-23 by LCs reduced the interaction between LCs and γδ T cells, thereby inhibiting γδ T cells and, in turn, IL-17 secretion, ultimately leading to improvements in psoriasis-like symptoms in a murine model [30]. Interestingly, in cancer patients with immune-related adverse events (irAE), the development of psoriasis is associated with the loss of epidermal CD1a^+^ LCs after treatment with immune checkpoint inhibitors (ICI). It is likely that the ICI-therapy aggravates a pre-existing psoriasis phenotype in some cancer patients with low numbers of LCs in the epidermis [31]. In another recent study, it was shown that TGFβ is important in maintaining functional LCs and memory T cells in the skin. Moreover, authors show that integrins avβ6 and avβ8, which are able to activate latent TGFβ, were increased in human keratinocytes from psoriatic skin, which might contribute to maintaining cutaneous inflammation [32]. We have shown increased numbers of epidermal LCs with predominant basal/suprabasal, as well as dermal location, in psoriatic skin lesions (Figure 2).

### 2.3. The Role of Langerhans Cells in Other Inflammatory Skin Pathologies

A pathology with clear LC involvement is dermatopathic lymphadenopathy. This disease starts in the skin lymph nodes, which drains the sites of chronic cutaneous diseases, ranging from infections to neoplasms. A recent study has shown in several dermatological disorders (i.e., mycosis fungoides, chronic inflammatory dermatoses, melanoma, squamous cell carcinoma and Kaposi sarcoma upon human immunodeficiency virus infection), the presence of three types of DC subsets in the paracortical regions of lymph nodes. These APCs were immunophenotypically identified as LCs (S100^+^, CD1a^high^, langerin^+^), interdigitating DCs (S100^+^, CD1a^low^, langerin^−^) and a minor DC subpopulation (S100^+^, CD1a^−^, langerin^−^). Moreover, the localization of langerin^+^ cells via IHC in the trabecular and medullary regions can contribute to ameliorating the differential diagnosis in LCH [33].

The increasing rate of infection with genus beta human papillomaviruses (HPV) [34], which has been shown to be involved in several cancers, including non-melanoma skin cancer (NMSC), raises concerns in the whole population. Ten years ago, the first case of HPV infection in patients diagnosed with epidermodysplasia verruciformis (EV), was reported. Interestingly, LCs are consistently lacking in HPV8^+^ skin samples from EV patients. It was shown that the differentiation-associated transcription factor CCAAT/enhancer binding protein β (C/EBPβ), which is a target of HPV8 E7 oncoprotein, critically controls the expression of the *CCL20* gene in keratinocytes. Thus, HPV8 E7 suppresses the C/EBPβ-inducible and constitutive expression of *CCL20,* and hinders LC migration [35].

LCs express a receptor that recognizes the presence of double-stranded DNA of microbial or host cellular origin, called absent in melanoma 2 (AIM2). AIM2 induces IL-1β via the activation of the inflammasome. In inflammatory skin disorders, such as psoriasis and atopic dermatitis, and in experimental wounds or chronic venous leg ulcers, the upregulation of AIM2 in LCs might significantly contribute to pathomechanisms by enhancing the production of inflammatory cytokines, such as IL-1β, able to activate surrounding keratinocytes [36].

The role of LCs in atopic dermatitis has been extensively studied and reviewed elsewhere [37,38,39,40].

## 3. Skin Cancers

Skin cancer is the most frequent solid tumor neoplasm worldwide and a significant challenge for clinicians and researchers [41]. Skin tumors can originate from the abnormal transformation of keratinocytes, as observed in non-melanoma skin cancer (NMSCs), whose two main forms are basal cell carcinoma (BCC) and squamous cell carcinoma (SCC), or from the abnormal transformation of melanocytes, which can evolve into cutaneous melanoma (CM) [42,43]. LCs are likely involved in the initial anti-cancer immune response of the skin.

The most studied skin pathology related to LCs is Langerhans cell histiocytosis (LCH). The pathogenesis of LCH is still a matter of debate, with some groups arguing for a non-cancerous proliferation of LCs, whereas others advocate a neoplastic process. However, LCH was recently designated as an oncological disease driven by the neoplastic transformation of LCs [44]. In line with this, BRAF-mutation-driven hematological neoplasia and thyroid carcinomas have been found in LCH. Moreover, mutations in BRAF-V600E proto-oncogene are combined with MEK and ERK phosphorylation, which strongly suggests that there is a neoplastic transformation of LCs in LCH [45]. A BRAFV600E mutation occurs in around half of the pediatric cases of LCH. Vemurafenib (VMF), an approved BRAF inhibitor for melanoma, was also approved for LCH [46,47,48]. Similar to other skin pathologies, where VMF is clinically used, in LCH, the development of resistance to these inhibitors in patients has turned research toward other mutations in LCH pathogenesis.

A recent work reported the first case of concomitant LCH and cutaneous melanoma exhibiting a BRAF V600E mutation. In this patient, a diffuse expression of CD1a, langerin/CD207, S100 protein, and BRAF (VE1) was found within LCH. Nevertheless, the link between the occurrence of melanoma and LCH in the same patient is unknown [49]. Furthermore, a recent case report of poly-neoplastic syndrome showed that in a patient with eight different cancers, five of them exhibited a BRAF V600E mutation (i.e., LCH, chronic lymphocytic leukemia, histiocytic sarcoma, melanoma, adenocarcinoma of the lung), in contrast to the other neoplasms (i.e., multiple myeloma, basal cell carcinoma, and papillary thyroid cancer). This mutation was not detected in the healthy tissue of the patient, clearly pointing to a somatic mutation specifically affecting cancer cells [50].

Somatic mutations in ARAF and MAP2K1, activating the RAS-RAF-MEK-ERK pathway [45] or the over-expression of p16(INK4a) and p21(CIP1/WAF1) [51] in the absence of a BRAF mutation in LCH, should polarize further investigation in order to develop more effective targeted therapies. It has been shown that the accumulation of mutations in the RAF/MEK/ERK pathway leads to an immature LC phenotype [52,53,54]. Therapies based on MEK inhibitors, such as trametinib, were successfully tested in LCH patients, showing that MEK1 mutations can be efficiently targeted in this disease [55].

**Figure 3:**
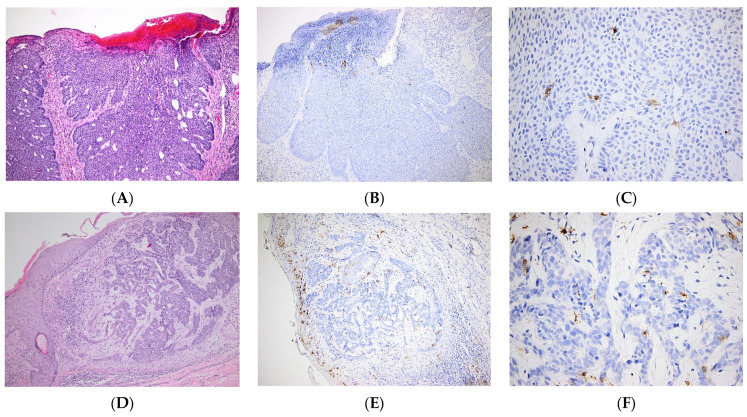
Langerhans cells in Basal cell carcinoma. (**A**–**C**). Nodular ulcerated basal cell carcinoma. (**A**). Tumor islands of basaloid cells with peripheral palisading. HE × 100. (**B**). Few Langerhans cells within the tumoral mass, most frequent in the superficial area, beneath the necrotic debris. CD1a × 100. (**C**). Very few Langerhans cells within the tumor islands. CD1a × 400. (**D**–**F**). Infiltrative basal cell carcinoma. (**D**) Cords and trabeculae of basaloid cells. HE × 100. (**E**). Relatively frequent Langerhans cells within the tumoral structures; numerous Langerhans cells within the suprajacent epidermis; small nodular foci of Langerhans cells within the peritumoral inflammatory infiltrate. CD1a × 100. (**F**). Relatively frequent Langerhans cells within the tumor, both intra-tumoral and in tumor stroma. CD1a × 400.

**Figure 4:**
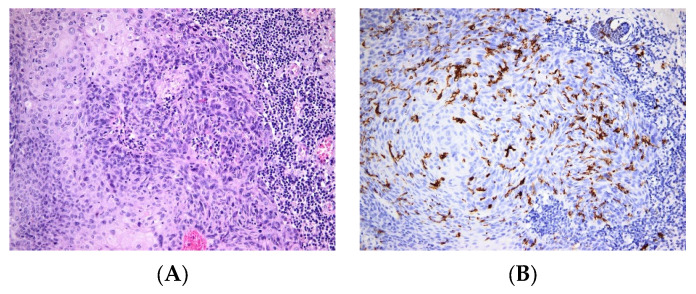
Langerhans cells in Squamous cell carcinoma. (**A**). Squamous cell proliferation with moderate cellular atypia; abundant peritumoral inflammatory infiltrate. HE × 200. (**B**). Numerous Langerhans cells present within the tumor mass with diffuse distribution in the tumoral structures. CD1a × 200.

In NMSCs, it was recently reported that the percentage of LCs in the epidermis correlates with the clinicopathological features of the skin tumor. Moreover, the percentages of LCs are higher in healthy epidermis when compared to the adjacent epidermis, flanking the tumoral tissue, and to the intratumoral tissue. Furthermore, the percentages of LCs are higher in BCC than in SCC [56]. In contrast, our data show very few LCs within the tumoral mass of BCC (Figure 3), as opposed to SCC, where LCs are relatively abundant (Figure 4). In a case report, the occurrence of skin SCC was associated with that of several carcinomas, including a Langerhans cell sarcoma (LCS). After the patient’s death, the presence of LCS metastasis was confirmed in several organs [57].

The majority of reports investigating the role of LCs in “classical” skin cancers have focused on melanoma. The reports identified several levels of LC involvement in melanoma genesis and in tumor progression. Non-invasive in vivo imaging of skin cancers includes a panel of new technologies that aid dermatologists in their diagnosis. Among them, reflectance confocal microscopy (RCM) is more and more broadly utilized [58]. However, RCM does not allow differentiating intraepidermal LCs from melanocytes, notably because LCs can simulate a pagetoid spread, similar to melanocytes [59]. Nevertheless, the use of CD1a as a specific marker of LCs is a powerful tool to distinguish both cell populations with precision [60].

**Figure 5:**
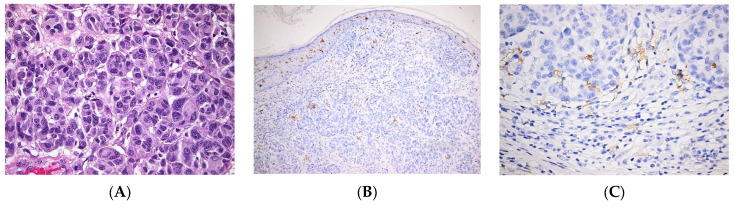

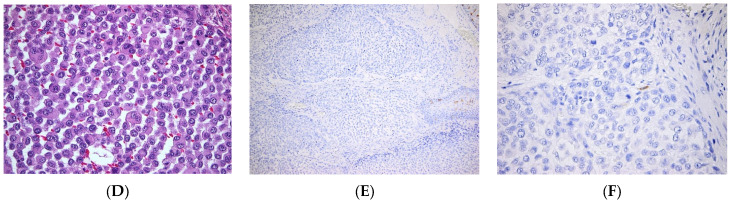
Presence of Langerhans cells in nodular melanoma with various phenotypic appearances. (**A**). Small nests of highly pleomorphic melanocytes. HE × 400. (**B**). Relatively frequent Langerhans cells within the tumor mass. CD1a × 100. (**C**). Numerous Langerhans cells present within the tumor nests and peritumoral inflammatory infiltrate. CD1a × 400. (**D**). Large nests of moderately pleomorphic melanocytes with marked discohesivity. HE × 100. (**E**). Absence of Langerhans cells within the tumor; several Langerhans cells observed in the hair follicle (positive control). CD1a × 100. (**F**). Absence of Langerhans cells within the tumor; one single positive cell observed in the inflammatory infiltrate. CD1a × 400.

Within CM tumor microenvironment (TME), the lymphocyte infiltrate shapes the anti-tumoral response. The majority of skin melanomas show evidence of T cell predominance infiltrating the tumor. Besides T cells, there are other immune cells that actively contribute to TME, such as LCs, macrophages, DCs, mast cells and natural killer cells. The efficacy of immune therapies in treating melanoma resides in the composition of the tumor immuno-microenvironment, and on the crosstalk between immune and tumor cells [61]. Intriguingly, some melanoma tumors have been shown to contain high amounts of LCs, whereas others are devoid of these cells (Figure 5). It is likely that molecular characteristics of LCs determine their ability to infiltrate TME. A recent work showed that one of these characteristics might be the expression of different TLRs in LCs [62].

Furthermore, we have shown that, in CM, the intra-tumoral inflammatory infiltrate can inform a patient’s prognosis. A good clinical prognosis is characterized by an infiltrate enriched in CD3^+^ T cells and LCs [63]. In patients, a poor prognosis correlates with a decreased CD4^+^/CD8^+^ T cell ratio in blood circulation. Importantly, we found that in advanced disease stages, the increase in this ratio is still associated with an overall better survival rate when compared to other groups. Mechanistically, IL-6 might govern the communication between LCs and T cells, and blood levels of this cytokine correlate with melanoma patients’ survival [63]. IL-6 has pleiotropic effects on inflammation, immune response and hematopoiesis. It is involved in the stimulation of antibody production and of effector T cell development, being a dual cytokine, with both anti and pro-inflammatory properties [64]. However, in TME, the activation of the IL-6-mediated STAT3 pathway mediates the inhibition of DC function and, in turn, dampens the T cell-mediated anti-tumoral response [65].

In sentinel lymph nodes (SLNs) harvested from CM patients, LCs display molecular features which favor immune tolerance. Indeed, langerin^+^ LCs localized in the T cell-rich area of SLNs express indoleamine 2,3-dioxygenase (IDO1) [66]. Moreover, IDO is strongly expressed when epidermal LCs are stimulated with IFN-γ [67]. In line with this, in melanoma SLNs, LCs display altered antigen presenting machinery (APM). LC HLA-class I APM components (i.e., Delta, LMP-7/10, TAP-1, Calnexin, Tapasin, β2-microglobulin and HLA-A,B,C) are lowly expressed in immature epidermal LCs and significantly increase upon activation. APM expression was found to negatively correlate with several pathological parameters, such as the melanoma Breslow’s thickness and the presence of metastases in SLNs. The expression of all HLA types (A, B, C) is lower in LCs from SLNs harvested from thick lesions than in thin/intermediate lesions; meanwhile, β2-microglobulin is over-expressed in melanoma positive SLNs when compared to negative ones. However, the overall results point towards the partial immunogenic capacity of LCs in CM [68].

IDO1 is a strong tolerogenic enzyme inducing immune peripheral tolerance and tumor-induced tolerance [69]. LCs express IDO1 in melanoma, emphasizing their involvement in the local immune-regulatory processes [70]. Four LC subsets have been identified in SLNs: (i) mature IDO1^−^CD83^+^LCs, (ii) immature IDO1^−^CD83^−^LCs, (iii) tolerogenic mature IDO1^+^CD83^+^LCs, and (iv) tolerogenic immature IDO1^+^CD83^−^LCs. The subset of tolerogenic immature LCs is significantly increased in metastatic SLNs. However, these immature SLN LCs are capable of upregulating CD83 when they are stimulated in vitro with inflammatory cytokines. These results open possibilities to design new immunotherapeutic approaches targeting LCs in melanoma [66].

Integrins avβ6 and avβ8 are expressed in keratinocytes and activate TGFβ. They have recently been shown to contribute to LC function in melanoma. Skin melanomas display an increased expression of integrin avβ8, which correlates with a greater Breslow depth and poor prognosis. Mechanistically, it seems that TGFβ, produced by keratinocytes overexpressing avβ6 and avβ8, retains CD8^+^ resident memory T cells and LCs in the epidermis [71]. Recently, it was postulated that high levels of avβ8 create a TGFβ-rich TME, thus promoting the accumulation of non-tumor antigen specific T cells and preventing effective anti-tumor immunity [32].

Various in vivo imaging technologies are currently being employed to better differentiate melanocytic lesions. Several years ago, third-harmonic generation (THG) microscopy was first used for the in vivo identification of tumoral melanocytes. Melanocytes give THG-bright dendritic-cell-like signals, hence allowing one to distinguish melanocytes from intratumor LCs [72]. Another new diagnostic imaging tool, namely RCM, is increasingly utilized for the diagnosis of skin cancer [58], owing to its capacity for non-invasively evaluating the cellular architecture of a lesion. Using RCM, four types of melanomas have been identified in which DCs have been shown to play a predominant role: (i) melanomas with thin Breslow index, (ii) melanomas with roundish melanocytes smaller than DCs and a thicker Breslow index, (iii) melanomas with a thick Breslow index at the time of diagnosis and exhibiting dermal proliferation, and (iv) a mixed type [73]. All these findings emphasize the involvement of LCs in skin cancers, by both contributing to skin inflammation, as well as to tumor immune evasion.

Table 1 outlines the major involvement of LCs in skin pathologies.

## 4. LCs as Therapeutic Targets

LCs and dermal DCs are the main cells which induce antigen-specific immunity in the skin. This property is exploited in vaccination, demonstrating that skin is an ideal route for vaccine delivery. Trans-cutaneous delivery of cancer vaccines is an important recent therapeutic tool. However, the stratum corneum is a physical obstacle for delivering antigens to the skin DCs upon vaccination. Protocols are currently being developed to circumvent this physical barrier. A recent strategy using a skin permeation enhancer, namely glyceryl monooleate (MO), could successfully deliver antigens to LCs and to dermal DCs, hence enriching the portfolio of vaccine adjuvants as a promising approach to eliminating tumors [76].

Developed almost 10 years ago, synthetic double-stranded (ds) RNA, namely polyriboinosinic acid-polyribocytidylic acid (poly(I:C)), mimics viral dsRNA and is a good immune-stimulator. Poly(I:C)-mediated immune activation depends on Toll-like receptor 3 (TLR3), and melanoma differentiation-associated gene-5 (MDA-5), for driving cell-mediated immunity and type I interferon response [77]. Pattern recognition receptor agonists, such as poly(I:C), can be adjuvant candidates for vaccination. Poly(I:C) was recently shown to activate LCs in skin explants or in cell cultures. In contrast to KCs, LCs express MDA5, rendering this molecule an attractive adjuvant for epicutaneous delivery of vaccines targeting LCs. Indeed, the in vitro treatment of LCs with poly(I:C) induced the mRNA of both IFN-α and IFN-β [78].

Furthermore, targeting glycan-coated antigens, such as C-type lectin receptors (CLRs) expressed by dermal DCs or langerin expressed by LCs, would enhance endocytosis and antigen processing. Moreover, simultaneous targeting of several DC subsets, including LCs, would endow a more robust and efficient T cell stimulation. With this aim, Duinkerken et al. have developed a branched polyamidoamine (PAMAM) dendrimer, serving as a scaffold for melanoma-specific gp100 peptides and a common ligand Lewis Y (LeY) for DCs and LCs [79]. DC-SIGN and Langerin, which are present on dermal DCs and LCs respectively, share a glycan binding specificity for LeY [80]. Their finding shows that CD1a^+^LCs and CD14^+^DCs can be simultaneously targeted, hence improving T cell specific activation and a CD8^+^ T cell-mediated anti-tumor response [79]. Recent studies have shown that melanoma antigens (e.g., gp100/MART-1), encapsulated in palmitoylated synthetic long peptides and alpha-galactosylceramide liposomes containing LeY, were better up-taken by monocyte-derived DCs, DCs and LCs, hence promoting a better activation of gp100-specific CD8^+^ T- and iNKT cells [81,82].

In 2021, Meneveau et al. took these results one step further in a pilot study in which LCs were targeted by a vaccine in melanoma patients. A vaccine, including twelve melanoma peptides combined with the granulocyte macrophage colony stimulating factor (GM-CSF) and various adjuvants, was administered into the dermis and subcutis of patients. The study has shown a measurable CD8^+^ T cell-mediated anti-tumoral response in over 80% of participants and an overall better survival of patients upon transdermal vaccination; this provided proof of the immunogenicity of LC-targeted vaccination via transdermal immunization [83].

With regard to clinical trials, in 2017 Chung et al. conducted a phase I vaccine trial using autologous LCs containing murine tyrosinase-related peptide-2 (mTRP2) mRNA. The study showed that 1–3 months after vaccination, antigen-specific CD4^+^ and CD8^+^ T cells were generated and able to produce detectable amounts of IFN-γ, IL-2, and TNF-α. This clinical trial demonstrated that manipulated autologous LCs were driving antigen-specific responses, including augmented cytokine secretion, cytolytic degranulation, and clonality in the T cell populations, which correlated with patients’ clinical outcomes [84].

Electrochemotherapy (ECT) was tested almost ten years ago for anti-tumor immunogenicity. Treatment of patients with ECT decreased the number of LCs in the treated tumor, suggesting an ECT-induced LC activation. In line with this, LCs up-regulated CCR7, a chemokine receptor involved in LC migration to the skin lymph nodes, and CD83, a maturation marker. Thus, these preliminary data suggest that LC emigration from the tumor to the skin draining lymph nodes is promoted by ECT [85,86].

**Figure 6:**
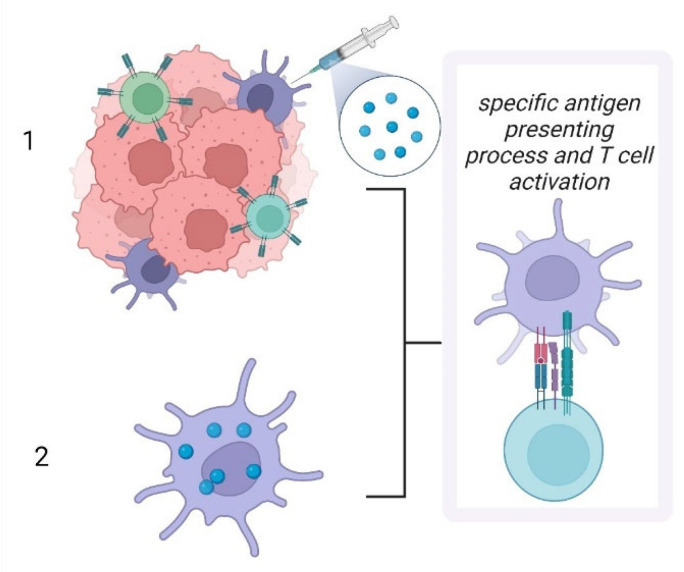
Therapeutic approaches using LCs. (1) In situ activation of LCs with specific antigenic peptides; (2) Ex vivo manipulation of LCs using specific antigenic peptides; both approaches induce antigen presentation enhancement and further T cell specific activation.

As summarized in Figure 6, therapeutic approaches targeting LCs have two main streams. The first, and probably the most applied protocol targets LCs in situ, whereas the second protocol, which is gaining momentum, is based on the ex vivo manipulation of LCs to generate an efficient anti-tumoral response.

Future research directions should focus on signaling pathways in LCs that maintain their immunogenic phenotype. Other important research subjects would be the mechanisms of LC maturation, senescence and migration in oncological contexts. Indeed, studying the influence of the microenvironment in shaping LC function could produce important information regarding the anti-tumor response. These studies would unravel new biomarkers, which will ultimately improve diagnosis, prognosis and new treatment efficacy in skin pathologies.

## 5. Conclusions

The skin immune system is a vast network of immune cells in which LCs play an important role; this is due to their function as APCs, in inflammatory, repair and autoimmune responses, as well as in tumorigenesis. LCs communicate with all the skin immune and non-immune cells (e.g., keratinocytes) through the secretion of an array of cytokines, chemokines, and growth factors, which eventually heightens the immune response. In psoriasis and atopic dermatitis, LCs are hyper-activated and induce or exacerbate adaptive immune responses, hence significantly contributing to disease pathogenesis. In skin cancers, if LCs can be activated through various therapeutical approaches, they can efficiently activate the anti-tumor immune processes. Therefore, in oncology, vaccination strategies targeting skin LCs might represent a valuable therapeutic avenue.

## Figures and Tables

**Table 1:** LC involvement in skin pathologies.

Pathology		LC Involvement
Bacterial and viral infections		Expression of pathogen-recognition receptors, secretion of cytokines, migration to skin draining lymph nodes to generate the adaptive immune response [2]
Wounds		Increased LC migration to skin draining lymph nodes mediated by enhanced expression of MICA B by keratinocytes [14,15]; TNF-mediated LC repopulation of the epidermis contributes to tissue remodeling [17,18]
Other skin pathologies	Psoriasis	Pro-inflammatory role of LCs via increased secretion of IL-23, over-expression of CD1a and increased interaction with γδT cells [27,30]
	Contact dermatitis	Uptake of allergens and immune response via antigen presentation to T cells [74]
	Atopic dermatitis	Activated LCs migrate to skin draining lymph nodes to prime Th2 T cells, increased expression of the high-affinity immunoglobulin (Ig)E receptor [37,38,39,40,75]
Skin cancers	BCC	LC are low in BCC [57]
	SCC	Various levels of LC infiltration in SCC, alternatively low [56] or high densities [54].
	Melanoma	Tolerogeneic vs. immunogenic role of LCs, infiltration of the tumor with LCs is a marker of good outcome [68]

## Data Availability

Not applicable.
